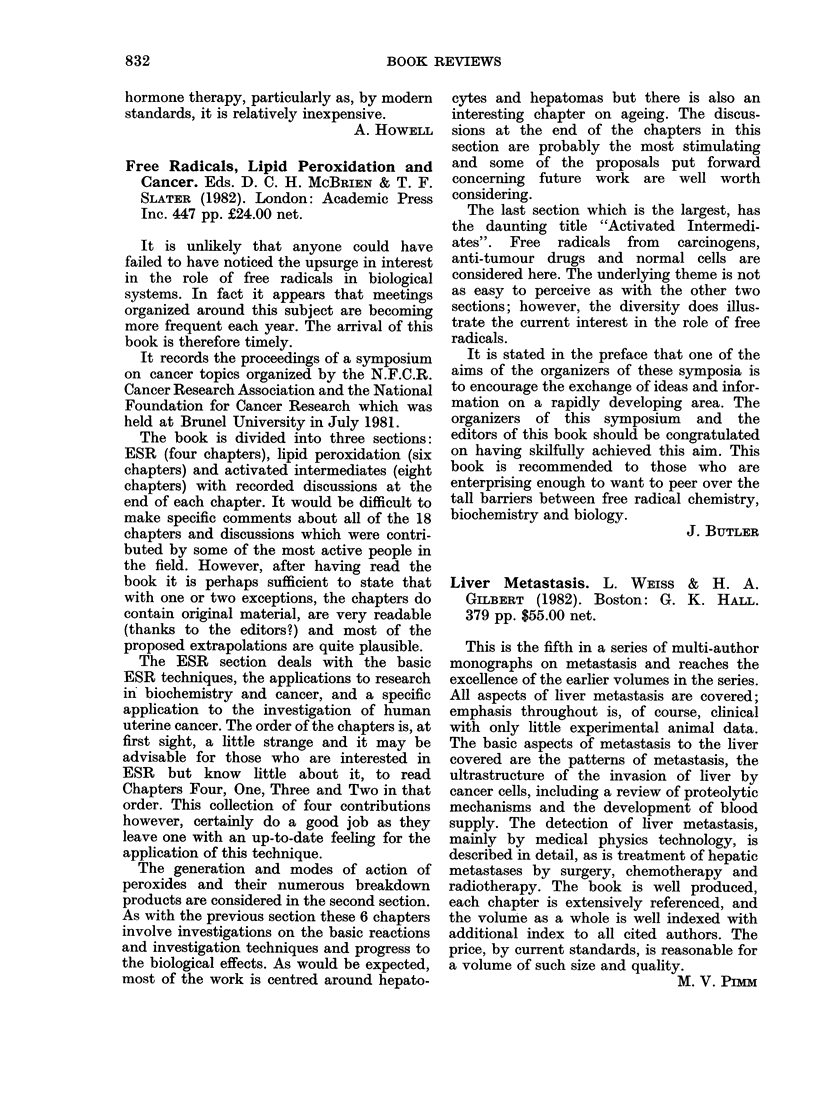# Liver Metastasis

**Published:** 1982-11

**Authors:** M. V. Pimm


**Liver Metastasis.** L. Weiss & H. A. Gilbert (1982). Boston: G. K. Hall. 379 pp. $55.00 net.

This is the fifth in a series of multi-author monographs on metastasis and reaches the excellence of the earlier volumes in the series. All aspects of liver metastasis are covered; emphasis throughout is, of course, clinical with only little experimental animal data. The basic aspects of metastasis to the liver covered are the patterns of metastasis, the ultrastructure of the invasion of liver by cancer cells, including a review of proteolytic mechanisms and the development of blood supply. The detection of liver metastasis, mainly by medical physics technology, is described in detail, as is treatment of hepatic metastases by surgery, chemotherapy and radiotherapy. The book is well produced, each chapter is extensively referenced, and the volume as a whole is well indexed with additional index to all cited authors. The price, by current standards, is reasonable for a volume of such size and quality.

M. V. Pimm